# Ilizarov treatment of humeral shaft nonunion in an antiepileptic drug patient with uncontrolled generalized tonic-clonic seizure activity

**DOI:** 10.1186/1749-799X-5-48

**Published:** 2010-07-28

**Authors:** Vasileios S Sioros, Marios G Lykissas, Dimitrios Pafilas, Panayiotis Koulouvaris, Alexandros N Mavrodontidis

**Affiliations:** 1 Department of Orthopaedic Surgery, University of Ioannina School of Medicine, Ioannina, Greece

## Abstract

Nonunion of the humeral shaft in patients with antiepileptic drug associated metabolic bone disorder constitute a challenging surgical problem difficult to treat due to seizure activity, osteoporosis, and poor stabilization options. We report a case of nonunion of the humeral shaft in an antiepileptic drug patient with uncontrolled generalized tonic-clonic seizure activity successfully treated with Ilizarov external fixator and a follow-up of 4 years.

## Background

Humeral shaft fractures account for approximately 1.3% of all fractures [1]. Approximately 1-15% of these fractures progress to nonunion [2-7]. Nonunion of the humeral shaft in patients with antiepileptic drug associated metabolic bone disorder constitute a challenging surgical problem difficult to treat due to seizure activity, osteoporosis, and poor stabilization options. Treatment options include internal fixation supplemented with cancellous bone graft, intramedullary nailing, free vascularized fibular graft, and Ilizarov circular frame fixation. At the hands of an expert surgeon, Ilizarov external thin-wire fixator can be a viable surgical option for the treatment of humeral shaft nonunion. We report a case of nonunion of the humeral shaft in an antiepileptic drug patient with uncontrolled generalized tonic-clonic seizure activity successfully treated with Ilizarov external fixator and a follow-up of 4 years.

## Case presentation

**Figure 1:**
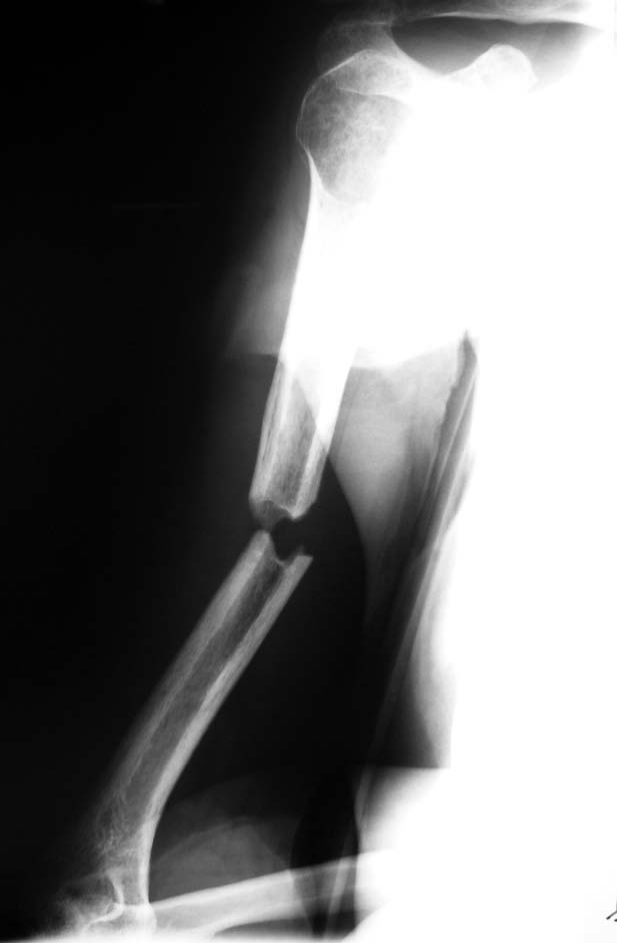
**Anteroposteriorradiograph of the right humerus of a 43-year-old man sustained a transverse diaphyseal fracture after a fall during a generalized tonic-clonic attack**.

**Figure 2:**
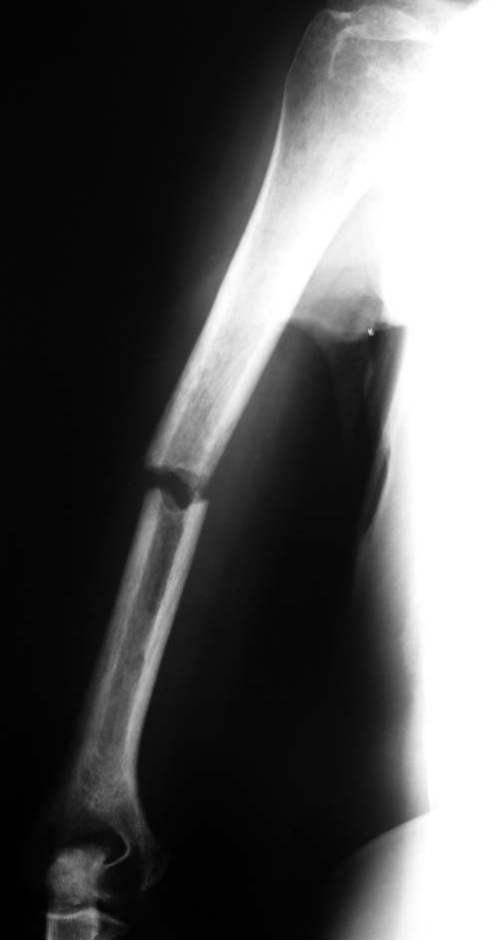
**Lateral view of the right humerus**.

A 43-year-old man was admitted to the emergency department after a fall during a generalized tonic-clonic seizure attack (grand mal). He sustained a closed transverse diaphyseal fracture of his right humerus (Figs. 1 &2). The patient suffered from epilepsy for the last 15 years and he was on carbamazepine (Tegretol CR 400 mg, Novartis, Greece) since then. Although well compliant with his treatment regimen, generalized tonic-clonic attacks occur almost once a week.

**Figure 3:**
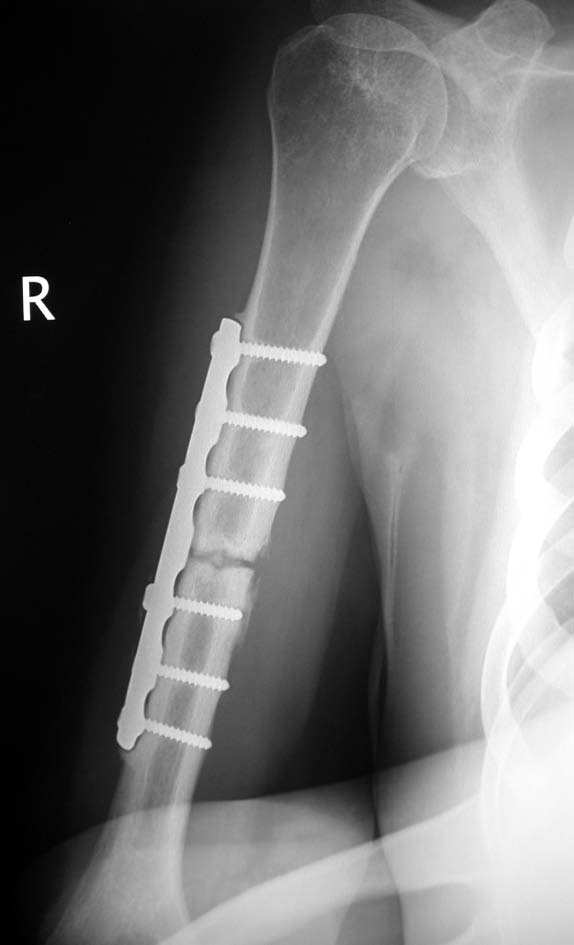
**Anteroposteriorradiograph of the right humerus showing atrophic nonunion of the humeral shaft 18 months after treatment with open reduction and internal fixation**.

**Figure 4:**
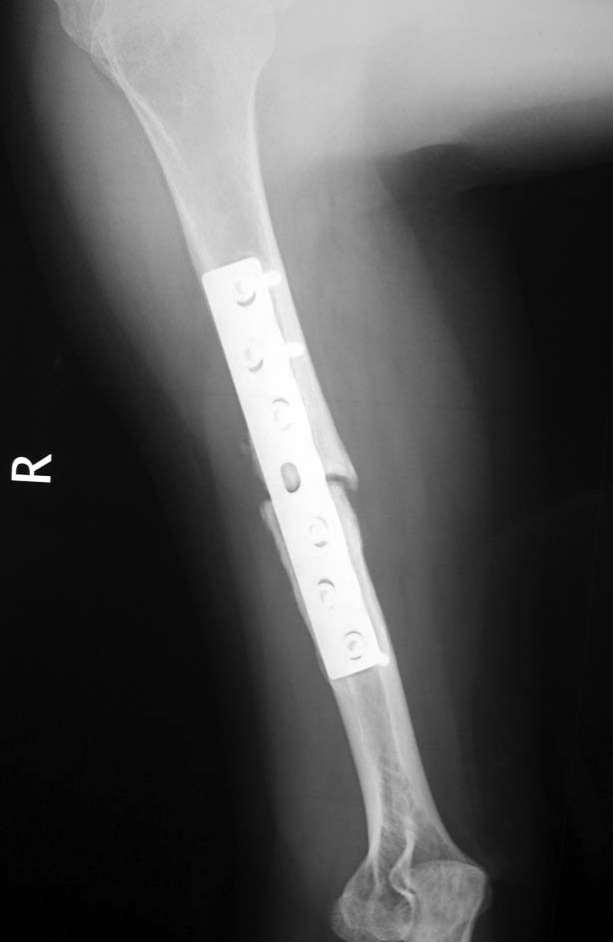
**Lateral view of the right humerus 18 months postoperatively**.

The fracture was initially managed by open reduction and internal fixation with plate and screws through an anterolateral longitudinal incision. Fixation was augmented with autologous bone graft obtained from the contralateral iliac crest. Eighteen months after surgery, radiographic evaluation revealed pseudarthrosis of the shaft of the humerus (Figs. 3 &4).

Exploration of the nonunion was performed under general anesthesia and using the prior incision. Prophylactic second generation cephalosporin antibiotic therapy was administered for 72 hours after surgery. The fracture site was opened and hardware materials were removed. Fibrous scar tissue and soft avascular bone was excised to expose fresh bleeding bone ends. The intramedullary canals were opened at the proximal and distal fragment. Following debridement, approximately a 1-cm segmental defect was measured. Specimens were sent for gram stain and microbiological analysis.

**Figure 5:**
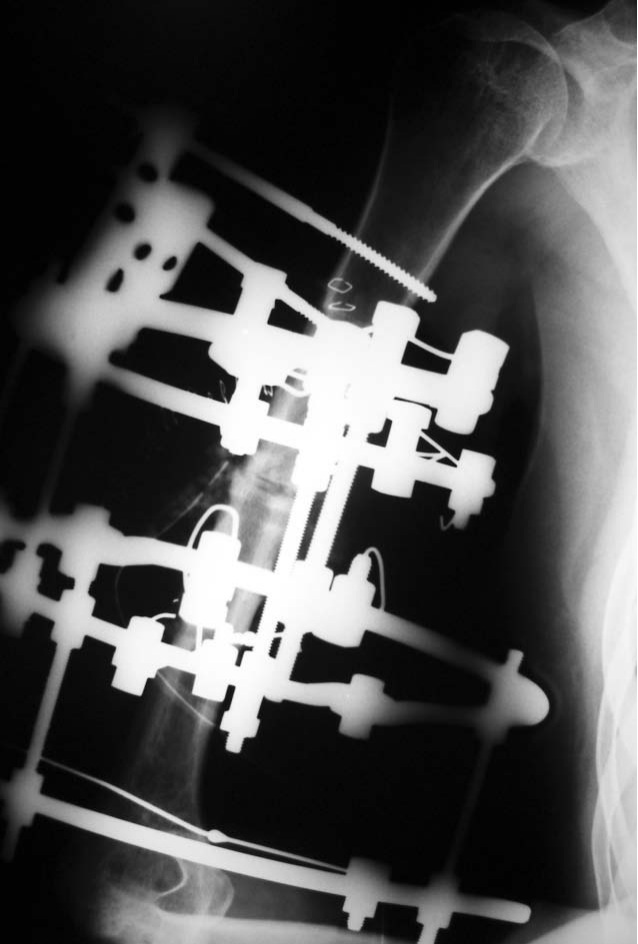
**Radiograph of the3-ring frame**.

**Figure 6:**
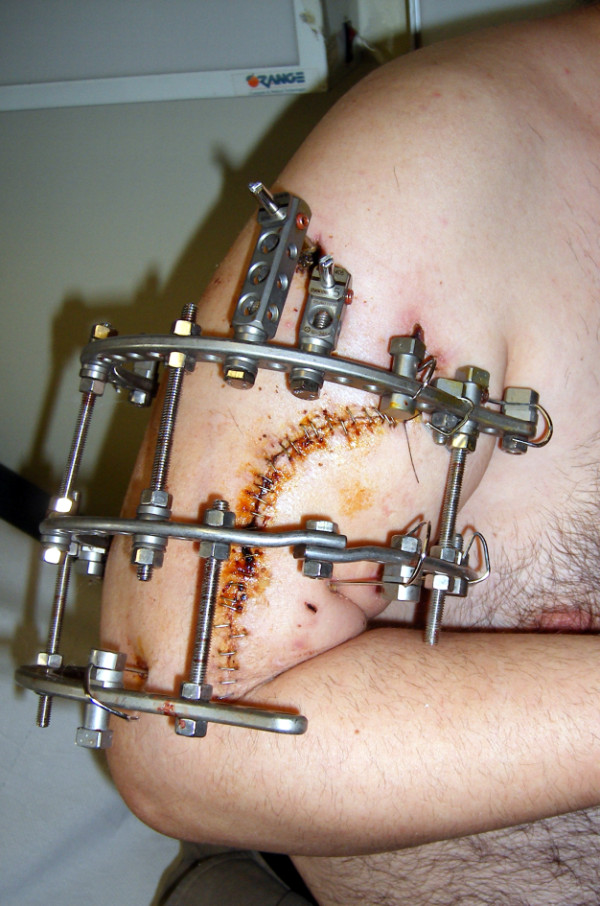
**Photograph of the same Ilizarov circular frame**. Note the proximal and distal 5/8 rings that facilitate active shoulder and elbow range of motion.

A 3-ring frame connected with 5 threaded rods was prefabricated using the left normal humerus as a template (Smith and Nephew plc, Memphis, Tennessee, U.S.A.). The fixator consisted of a 2-ring frame (full ring proximal and 5/8 ring distal) placed distally and a 5/8 1-ring frame placed proximally to the fracture site (Figs. 5 &6). The proximal and distal rings were not circular to facilitate active shoulder and elbow range of motion. Four thin wires (1.8 mm) with olives for both the distal frames and 2 thin wires (1.8 mm) with olives for the proximal frame were used, while 2 half pins (6.0 mm) were placed proximally in the mid-shaft of the humerus. Acute shortening of 1.0 cm via the Ilizarov fixator with immediate bone-to-bone contact at the nonunion site was then performed. The procedure was accomplished under fluoroscopic guidance. The radial nerve was explored in order to avoid nerve injury during wire insertion. Autologous cortico-cancellous bone graft harvested from the contralateral ilium was applied to the nonunion. The total operating time was 120 minutes.

Immediately after surgery the arm was placed in a sling for 6 weeks. From the first morning after surgery, joint mobilization of the shoulder and elbow was started as tolerated. In order to better control seizure activity, levetiracetam (Keppra 1000 mg, UCB Pharma S.A., Belgium) was added in the anticonvulsant therapy. The patient was instructed in pin care cleaning and hygiene and discharged from the hospital 5 days after surgery. Pin-tract infection was noticed in two skin/pin contacts which were treated with oral antibiotics (second generation cephalosporin) for one week.

**Figure 7:**
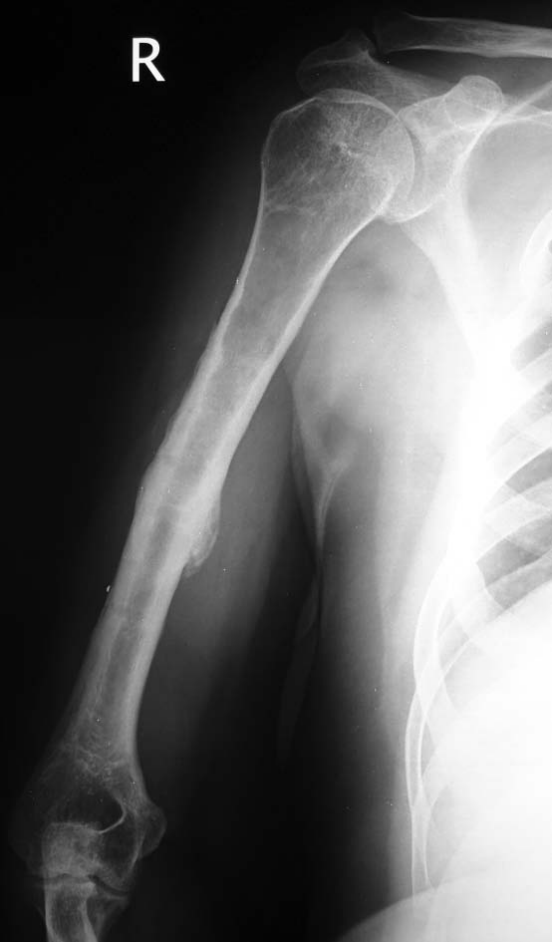
**Anteroposteriorradiograph of the humeral fracture 4 years after surgery**. Union was achieved 4.5 months after initial application of the frame.

**Figure 8:**
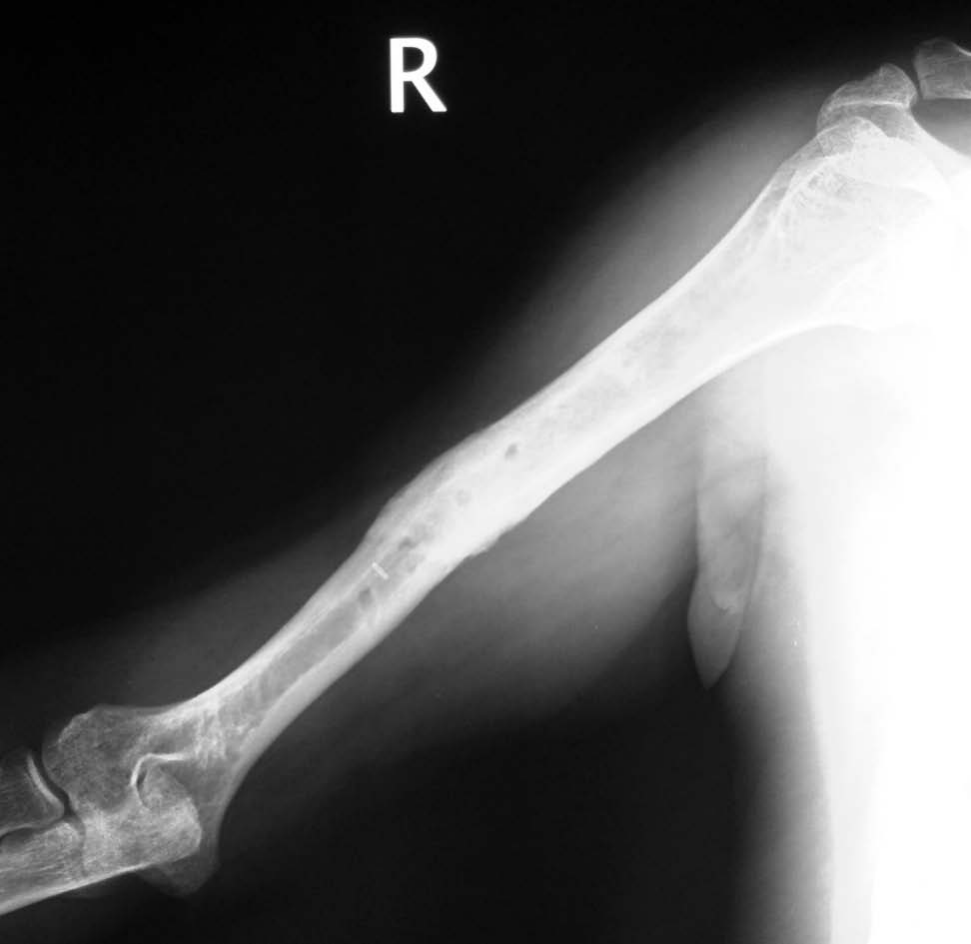
**Lateral view of the humeral fracture 4 years postoperatively**.

Antero-posterior and lateral radiographs demonstrated uncomplicated fracture healing at 18 weeks. The Ilizarov frame was removed at 24 weeks without anesthesia in the outpatient department. No protective immobilization was used after frame removal. At the most recent follow-up, 4 years postoperatively, the alignment of the humerus was anatomic and full range of motion was obtained at both the shoulder and elbow joint (Figs. 7 &8). The patient was very satisfied with his treatment and had returned to his previous activities.

## Discussion

Decreased bone density has been well documented in patients with epilepsy [8]. The occurrence of fractures in these patients is increased twofold to sixfold compared with than that expected in nonepileptic population [9]. In a comparative study of 202 institutionalized patients with epilepsy the frequency of fractures of the humerus was increased fourfold compared with a normal population [10]. The relative risk for humeral fractures is most increased in patients more than 45 years of age [11]. Seizure activity may cause fractures, usually vertebral compression fractures, as a result of spine hyperflexion during extreme muscular contractions [12]. Bilateral posterior fracture dislocation of the shoulder is highly indicative of seizure [13]. Trauma or fall during tonic-clonic, tonic, or atonic attack is also associated with fracture of the humerus along with fracture of the hip, ankle, and wrist [10,11]. Repetitive, uncontrolled seizure activity, especially tonic-clonic attacks, as in our case, may also adversely affect the process of fracture healing, making the management of such fractures a challenging surgical problem.

Antiepileptic drugs have been categorized as independent risk factors for decrease of bone mineral density regardless of patient's age, gender, and period of treatment [14]. Their role in bone loss is thought to be multifactorial. Conventional antiepileptic drugs, such as carbamazepine, phenytoin, and phenobarbital, are potent hepatic mixed-function oxidase (CYP450) inducers [15]. Valproic acid is a CYP450 inhibitor. Pregnabe × receptor (PXR), a transcriptional regulator of CYP450, mediates the adverse effect on bone metabolism of both CYP450 inducers and inhibitors through stimulation of vitamin D catabolism and inhibition of 25-hydroxylation of vitamin D [16]. The effect of antiepileptic drugs on bone mineral density is also mediated by Vitamin D receptor (VDR) gene, an important regulator of osteoclastic activity [17]. In turns, vitamin D catabolism results in decreased calcium absorption across the small intestine, hypocalcemia, and secondary hyperparathyroidism [18]. It has also been demonstrated that CYP450 exhibits antiproliferative and antidifferentiation effects on osteoblasts [19].

The deterioration of bone metabolism caused by conventional antiepileptic drugs highlights the role of these agents both in pathogenesis of special type of fractures and the need of vitamin D and calcium supplementation in this patient population [20]. The induction of bone loss by conventional antiepileptic drugs also emphasizes the need of special techniques to treat difficult cases, such as fracture nonunion.

The incidence of nonunion of humeral shaft fractures after both conservative and surgical management is reported to be as high as 1-15% [2-7]. Failure to unite after surgical management of diaphyseal fractures of the humerus could be multifactorial. Factors that may play a role in nonunion include inadequate fracture fixation, osteopenia/osteoporosis, infection, devitalization of bone, and poor contact between the fracture segments. Most nonunions of the humerus are associated with angulation, displacement, over-riding, limb shortening, and osteopenia. Treatment options include internal fixation supplemented with cancellous bone graft, intramedullary nailing, free vascularized fibular graft, and Ilizarov circular frame fixation. Locking plates and dual plating have also been proposed as alternatives in cases of nonunion of the humerus with poor bone stock.

Rigid internal fixation with plating is considered as the "gold standard" for the management of humeral shaft nonunion with union rates approaching 100% [21]. Augmentation with autologous bone graft is recommended, especially in atrophic type of nonunions, representing the 70-90% of all cases [21]. Atrophic aseptic nonunion of the humeral shaft after failure of surgical management, as in our patient, is characterized by poor bone quality. Further decrease in the bone mineral density, secondary to anticonvulsant bone disease, makes internal fixation less stable than in normal bone. Further complications after open reduction and internal fixation in a previous surgically treated humerus include difficult dissection in a scarry tissue environment with risk for radial nerve iatrogenic injury approaching 4% [22]. Superficial or deep infection following conventional methods of internal fixation is reported as high as 6.7% [22].

If intramedullary nailing is selected for the management of diaphyseal fractures of the humerus, nonunion is reported in a higher rate than plating, ranging from 0 to 33% [23,24]. Exchange nailing in cases of nonunion of the diaphysis femur or tibia is a viable method for achieving union. However, humeral shaft fractures complicated by nonunion cannot achieve union after reaming and exchange nailing [24]. This can easily be explained biomechanically by the absence of axial loading in the humerus and the presence of greater torsional and distractive forces than in tibia or femur [25]. Further drawbacks following intramedullary nailing include shoulder or elbow stiffness, depending on the point of insertion, radial nerve palsy, disruption of the endosteal blood supply, and fracture instability if the nail remains unlocked [26]. According to some authors, higher union rates can be achieved if the intramedullary nail is locked [21].

Ilizarov technique has been successfully used for the management of nonunion of the humeral shaft [27,28]. It is a very promising method because it is minimally invasive with low intraoperative blood loss, and minimal patient discomfort. It provides stable fixation, prompt postoperative elbow and shoulder mobilization, and has no major complications. It gives postoperative capability for malalignment correction and, at the hands of an expert, Ilizarov external fixation is not time consuming [28]. It appears that the Ilizarov apparatus is superior to conventional fixation methods, especially in patients with severe bony deformity, limb shortening, and bone loss [29]. In such cases, callus formation can be stimulated by controlled oscillating compression and distraction [5,25]. Long-lasting nonunion may lead to local osteoporosis which is different from osteoporosis due to old age. When severely compromised local bone due to disuse is associated with metabolic bone disorder, internal fixation is technically demanding and plate loosening often occurs. In our patient, severe osteoporosis due to local and systemic factors was accompanied by mechanical instability of the osteosynthesis because of the frequent tonic-clonic seizure activity. The Ilizarov external fixator was the only system that could simultaneously provide stable fixation in an osteoporotic bone, externally controlled compression, and interfere dynamically with repetitive seizures. Ilizarov does not support the use of bone grafting for the management of nonunions. However, autologous bone graft obtained from the iliac crest was used in our patient with atrophic nonunion in order to stimulate the biology of the nonunion site, speed the bone healing, and minimize the fixation time.

Ilizarov technique may involve the risk of pin-tract infections most of which can be treated by administration of antibiotics, as in our case. Other disadvantages include re-fracture following frame removal, limb shortening, radial nerve palsy, and patient discomfort because of the weight of the device and impingement of the frame on the chest. Re-fracture can be prevented with the use of a plastic brace after frame removal. Limb discrepancy of 3 to 4 cm is generally well tolerated and further shortening of the upper extremity can be managed by lengthening the humerus with a new Ilizarov frame in a later stage. Nerve injury during placement of the transosseous wires can be avoided by reducing the amount of paralytic agents given and looking for motor flickers to the wrist, hand or fingers. In order to allow early shoulder and elbow mobility and minimize the frame interference with daily activities, a semicircular proximal and distal ring should be used.

## Conclusions

The management of humeral shaft nonunion in antiepileptic drug patients offers a different challenge. In such cases, Ilizarov external fixator is an adequate treatment option that surgeon should always have in mind. It provides stable fixation, prompt postoperative mobilization, and has no major complications. It gives postoperative capability for malalignment correction and, at the hands of an expert, Ilizarov external fixation is not time consuming. When conventional antiepileptic drugs are used, vitamin D and calcium supplementation are recommended for prophylaxis and treatment of bone loss.

## Consent

Written informed consent was obtained from the patient for publication of this case report and any accompanying images. A copy of the written consent is available for review by the Editor-in-Chief of this journal.

## Competing interests

There are no competing interests; this is a basic academic research initiative.

## Authors' contributions

All authors contributed equally to this work. MGL and VSS participated in the design of the study and drafted the manuscript. ANM, DP, and PK conceived of the study and participated in its design and coordination.

Marios G. Lykissas has had the main responsibility for the study and manuscript preparation. All authors read and approved the final manuscript.

## References

[B1] Brinker MR O'Connor DP The incidence of fractures and dislocations referred for orthopaedic services in a capitated population J Bone Joint Surg Am 2004 86 290 7 14960673

[B2] Borus TA Yian EH Karunakar MA A case series and review of salvage surgery for refractory humeral shaft nonunion following two or more prior surgical procedures Iowa Orthop J 2005 25 194 9 16089097 PMC1888765

[B3] Durbin R Gottesman MJ Saunders KC Hackthal stacking nailing of humeral shaft fractures. Experience with 30 patients Clin Orthop Relat Res 1983 179 168 74 10.1097/00003086-198310000-00024 6617010

[B4] Healy WL White GM Mick CA Brooker AF Jr Weiland AJ Nonunion of the humeral shaft Clin Orthop Rel Res 1987 219 206 13 3555925

[B5] Jupiter JB von Deck M Ununited humeral diaphyses J Shoulder Elbow Surg 1998 7 644 53 10.1016/S1058-2746(98)90016-7 9883429

[B6] Marti RK Verheyen CC Besselaar PP Humeral shaft nonunion: evaluation of uniform surgical repair in fifty-one patients J Orthop Trauma 2002 16 108 15 10.1097/00005131-200202000-00007 11818806

[B7] Ring D Kloen P Kadzielski J Helfet D Jupiter JB Locking compression plates for osteoporotic nonunions of the diaphyseal humerus Clin Orthop Relat Res 2004 425 50 4 10.1097/01.blo.0000131484.27501.4b 15292787

[B8] Khanna S Pillai KK Vohora D Insights into liaison between antiepileptic drugs and bone Drug Discov Today 2009 14 428 35 10.1016/j.drudis.2009.01.004 19187798

[B9] Mattson RH Gidal BE Fractures, epilepsy, and antiepileptic drugs Epilepsy Behav 2004 5 Suppl 2 S36 40 10.1016/j.yebeh.2003.11.030 15123010

[B10] Desai KB Ribbans WJ Taylor GT Incidence of five common fracture types in an institutional epileptic population Injury 1996 27 97 100 10.1016/0020-1383(95)00189-1 8730381

[B11] Persson HBI Alberts KA Farahmand BY Tomson T Risk of extremity fractures in adult outpatients with epilepsy Epilepsia 2002 43 768 72 10.1046/j.1528-1157.2002.15801.x 12102682

[B12] Vasconcelos D Compression fractures of the vertebrae during major epileptic seizures Epilepsia 1973 14 323 8 10.1111/j.1528-1157.1973.tb03967.x 4270321

[B13] Elsberger ST Brody G Bilateral posterior shoulder dislocations Am J Emerg Med 1995 13 331 2 10.1016/0735-6757(95)90213-9 7755831

[B14] Khanna S Pillai KK Vohora D Insights into liaison between antiepileptic drugs and bone Drug Discov Today 2009 14 428 35 10.1016/j.drudis.2009.01.004 19187798

[B15] Patsalos PN Fröscher W Pisani F van Rijn CM The importance of drug interactions in epilepsy therapy Epilepsia 2002 43 365 85 10.1046/j.1528-1157.2002.13001.x 11952767

[B16] Collins N Maher J Cole M Baker M Callaghan N A prospective study to evaluate the dose of vitamin D required to correct 25-hydroxyvitamin D levels, calcium and alkaline phosphatase in patients at risk of developing antiepileptic drug-induced osteomalacia Q J Med 1991 286 113 22 1851568

[B17] Takasu H Sugita A Uchiyama Y Katagiri N Okazaki M Ogata E Ikeda K c-Fos protein as a target of anti-osteoclastogenic action of vitamin D, and synthesis of new analogs J Clin Invest 2006 116 528 35 10.1172/JCI24742 16424941 PMC1332025

[B18] Foxa SW Lovibond AC Current insights into the role of transforming growth factor-b in bone resorption Mol Cell Endocrinol 2005 243 19 26 10.1016/j.mce.2005.09.008 16219413

[B19] Feldkamp J Becker A Witte OW Scharff D Scherbaum WA Long-term anticonvulsant therapy leads to low bone mineral density-evidence for direct drug effects of phenytoin and carbamazepine on human osteoblast-like cells Exp Clin Endocrinol Diabetes 2000 108 37 43 10768830 10.1055/s-0032-1329213

[B20] Pack AM The association between antiepileptic drugs and bone disease Epilepsy Curr 2003 3 91 5 10.1046/j.1535-7597.2003.03306.x 15309069 PMC321183

[B21] Tomić S Bumbasirević M Lesić A Mitković M Atkinson HD Ilizarov frame fixation without bone graft for atrophic humeral shaft nonunion: 28 patients with a minimum 2-year follow-up J Orthop Trauma 2007 21 549 56 10.1097/BOT.0b013e31814612c8 17805022

[B22] Hsu TL Chiu FY Chen CM Chen TH Treatment of nonunion of humeral shaft fracture with dynamic compression plate and cancellous bone graft J Chin Med Assoc 2005 68 73 6 10.1016/S1726-4901(09)70138-8 15759818

[B23] Hems TE Bhullar TP Interlocking nailing of humeral shaft fractures: the Oxfo experience 1991 to 1994 Injury 1996 27 485 9 10.1016/0020-1383(96)00056-3 8977834

[B24] Lin J Hou SM Antegrade locked nailing for humeral shaft fractures Clin Orthop Relat Res 1999 365 201 10 10.1097/00003086-199908000-00025 10627704

[B25] Lammens J Bauduin G Driesen R Moens P Stuyck J De Smet L Fabry G Treatment of nonunion of the humerus using the Ilizarov external fixator Clin Orthop Relat Res 1998 353 223 30 10.1097/00003086-199808000-00026 9728178

[B26] Cierny G III Mader JT Approach to adult osteomyelitis Orthop Rev 1987 16 259 72 3454938

[B27] Patel VR Menon DK Pool RD Simonis RB Nonunion of the humerus after failure of surgical treatment. Management using the Ilizarov circular fixator J Bone Joint Surg Br 2000 82 977 83 10.1302/0301-620X.82B7.10180 11041585

[B28] Beris AE Lykissas MG Sioros V Mavrodontidis AN Korompilias AV Femoral periprosthetic fracture in osteoporotic bone after a total knee replacement. Treatment with Ilizarov external fixation J Arthroplasty 2010 in press 20097034 10.1016/j.arth.2009.10.009

[B29] Kocaoğlu M Eralp L Tomak Y Treatment of humeral shaft non-unions by the Ilizarov method Int Orthop 2001 25 396 400 10.1007/s002640100295 11820451 PMC3620784

